# Factors Influencing Pain Management Practices in People With Parkinson's Disease: A Qualitative Descriptive Study

**DOI:** 10.1155/padi/1231126

**Published:** 2025-08-01

**Authors:** Anthony Mezzini, Saravana Kumar, Sue Sharrad, Joanne Harmon, Marion Eckert

**Affiliations:** ^1^Rosemary Bryant AO Research Centre, Clinical and Health Sciences, University of South Australia, Adelaide, South Australia, Australia; ^2^IIMPACT in Health, Allied Health and Human Performance, University of South Australia, Adelaide, South Australia, Australia

## Abstract

**Background:** While Parkinson's disease (PD) is primarily recognized for its motor symptoms, several non-motor symptoms may also be present. Among these, pain is one of the most common and debilitating, arising from complex neurophysiological mechanisms that often interact with motor symptoms and comorbidities, leading to a diverse range of clinical presentations. Although a variety of pharmacological and nonpharmacological therapies are used to manage pain in PD, the factors influencing treatment practices remain underexplored, particularly within the Australian healthcare context. This study, therefore, aimed to explore, from the patients' perspective, factors that influence pain management practices among people with PD living in Australia.

**Methods:** A qualitative descriptive research methodology using a maximum variation sampling strategy was used to recruit people with PD, living in Australia. Data were collected using individual, semistructured interviews and thematically analyzed.

**Results:** 18 participants shared their perspectives on the factors that influenced their pain management practices. Thematic analysis of interview data resulted in four themes: (1) recommendations from trusted sources; (2) explorative experimentation and solution seeking; (3) intervention and service provider characteristics; and (4) personal beliefs and abilities; and several subthemes. These findings highlight the complex nature of therapeutic decision-making from the perspective of people with PD, underscoring the interaction between external and internal influences.

**Conclusion:** There is complexity and nuance in how people with PD make decisions about managing their pain. External and internal factors seem to influence therapeutic decision-making, while also highlighting notable gaps in the provision of PD pain care services. Understanding these complexities will be critical in developing accessible, effective, and patient-centered approaches to pain management within this population.

## 1. Introduction

Globally, Parkinson's disease (PD) is the second most common neurodegenerative disorder, surpassed only by Alzheimer's disease [1]. In Australia, it has been estimated that in 2014 there were 69,208 people living with PD (294 per 100,000) and that the total economic cost to the community (financial costs and burden of disease) was over $9.9 billion [2]. The disease is characterized by several motor (i.e., bradykinesia, rigidity, tremor, and impaired postural stability) [3, 4] and non-motor (i.e., depression, anxiety, sleep and gastrointestinal disorders, fatigue, and pain) [5, 6] symptoms. Among the symptoms of PD, pain often emerges early in the disease course [7, 8], but may also present at any disease stage [9, 10]. A systematic review by Broen et al. [11], examining pain prevalence in PD, reported a frequency range of 40%–85%, with an average of 67.6%. This rate has consistently been found to be higher than observed in the general population, as shown in multiple case-controlled studies [12–16]. In the early stages of PD, pain has been rated as the most troublesome non-motor symptom [17] and is a leading driver of reduced health-related quality of life [18–20]. In some cases, the pain is so severe and unmanageable, that it overshadows the motor symptoms of the disease [21]. In addition, pain in PD has also been shown to interact with other non-motor symptoms. Research using case–control study designs has found that people with PD that reported pain symptoms were more depressed [12, 15, 22, 23], experienced elevated levels of anxiety [12, 15, 24], and had poorer sleep quality [24, 25], compared to those without pain.

Research into PD pain mechanisms has revealed that neurodegenerative changes specific to the disease affect cerebral, spinal, and peripheral nociceptive pathways [26, 27]. These pathophysiological alterations can interact with comorbid conditions (such as osteoarthritis) and motor symptoms of PD (such as bradykinesia and rigidity), leading to a wide range of clinical manifestations [28–31]. To enhance the identification and management of pain in PD, various classification frameworks have been developed to group the diverse pain presentations into specific subtypes [12, 30–34]. Among the most widely referenced is the system proposed by Ford [31], which categorizes pain into five subtypes based on the etiology of pain symptoms: musculoskeletal, radicular/peripheral neuropathy, dystonic, central pain, and akathisia. Musculoskeletal pain and dystonic pain are reported as the most prevalent subtypes according to etiological classification studies [35–37]. Notably, many people with PD experience multiple pain subtypes simultaneously, underscoring the multifaceted and variable nature of pain in this population [14, 22, 35].

A range of therapies are available for the treatment of PD pain [38, 39], and several quantitative studies have explored their use among PD patients [11, 21, 40]. Buhmann et al. [21] conducted a cross-sectional study of PD patients (*n* = 178) and found that 74% of respondents with pain had received some form of pharmacological (other than dopaminergic) or nonpharmacological pain therapy at some point. The mostly commonly utilized therapies included physiotherapy (61%), analgesics (54%), and massage (36%). The factors influencing pain management practices in this population, however, have been less extensively studied. In a qualitative study of pain management experiences from the perspective of people with PD and carers (*n* = 125), Naisby et al. [41], thematically analyzed responses to open-ended survey questions and found that a low response rate to therapies, an unwillingness of participants to utilize pain medications, and the preference to self-manage the condition may be some of the factors influencing pain management practices. While the findings are novel, the transferability of these findings to other jurisdictions may be limited. Variations in clinical approaches to managing conditions such as PD and chronic pain across different geographic regions have been reported in the literature [42–44]. Moreover, the lack of dedicated clinical practice guidelines for the treatment of pain in PD [45], combined with studies highlighting cross-cultural differences in pain-related beliefs, coping mechanisms, and the extent of pain catastrophizing [46], further suggests PD pain management practices may vary across regions. In addition, relying solely on open-ended survey responses may not fully capture the complex factors affecting patients' treatment choices. Understanding these factors is clinically relevant to pain care providers, as it can aid in personalizing patient discussions and educational strategies, enhance treatment adherence, reduce the impact of misinformation, and ultimately, improve patient outcomes. Therefore, the purpose of this study was to explore the factors influencing the pain management practices of people with PD living in Australia.

## 2. Methods

### 2.1. Methodology

This was a qualitative descriptive study [47]. A descriptive approach was deemed appropriate to address the aims of the study because the focus is not on increasing theoretical or conceptual understanding, such is the case with other forms of qualitative research (i.e., phenomenology and grounded theory), but rather, contributing to clinical practice change and improvement [48, 49]. The study was conducted following the Standards for Reporting Qualitative Research (SRQR) guidelines [50].

### 2.2. Study Participants and Selection Procedures

To ensure a wide range of perspectives were captured, maximum variation sampling [51] was used to recruit participants. Participants were recruited from a pool of participants (*n* = 52) involved in a cross-sectional online survey that investigated pain management practices in people with PD conducted by the authors in 2023/24 and indicted they would be willing to participate in future research. Potential participants were contacted via email that included information about the study and a brief questionnaire detailing demographic (age, sex, and residential postcode) and clinical (disease stage and primary treating medical practitioner) characteristics. Participants were selected from those that responded to the email (*n* = 26) to reflect different demographic and clinical characteristics. To qualify for the present study, participants must have indicated in the original survey that they met the following criteria: (a) a diagnosis of PD by a neurologist or movement disorder specialist; (b) self-reported PD pain symptoms in the past month; (c) Australian citizenship or permanent residency and currently living in Australia; and (d) aged 18 years or older. In the survey, PD pain symptoms were defined as 10 specific pain types identified in the literature as being more prevalent in people with PD than in the general population [29–31, 52–54]. These included: dystonic pain, dyskinetic pain, temporomandibular pain, musculoskeletal pain, abdominal visceral pain, radicular neuropathic pain, peripheral neuropathic pain, restless legs syndrome, nocturnal akinesia, and central Parkinson's pain. For each pain type, participants were provided with a detailed description of the symptomatology based on previous reports [30, 32, 53, 55–60] and asked whether they had experienced these symptoms within the past month.

Given the absence of established criteria for determining sample size in descriptive qualitative research [49], a pragmatic approach was adopted, with data collection continuing until novel insights relevant to the study aims became sufficiently rare that additional interviews were unlikely to meaningfully impact the study results.

### 2.3. Data Collection

Data were collected between March and April 2024 via individual, semistructured interviews. A customized interview guide using open-ended questions was developed for this study by the corresponding author (AM)—an accredited exercise physiologist and PhD candidate—in consultation with members of the research team (SK, SS, and JH) who have combined expertise in qualitative research, pain management, and PD. This method of data collection was chosen because it allows the participant to focus on the issues that are meaningful to them, facilitating an in-depth and individualized understanding of their experiences and perspectives [61]. The interview guide compromised two main sections, featuring a set of open-ended questions at two levels: main topics which broadly directed the interview, and follow-up questions for explorative purposes. Section (A) focused on the factors influencing pain management practices, and Section (B) on the perceptions of the provision of pain care and support services. The results from Sections (B) will be reported in another study and, therefore, are not discussed further. The interview guide was reviewed by an expert in qualitative research external to the research team to assess its appropriateness and comprehensiveness in relation to the study aims. For reliability and continuity, all interviews were conducted by one member of the research team (AM). The interview was divided into two parts: an introduction and the interview questions. To build rapport and promote open dialogue with participants, the introduction included information about the interview process, an overview of the interviewer's background, assurances of confidentiality and anonymity, and encouragement to speak freely and share “their story.” In the second part of the interview, departures from the interview guide were made, where appropriate, to maintain dialogue relevancy and flow. Interviews typically lasted between 45 min and 1 h and were conducted either face-to-face or via video conferencing (i.e., Zoom, WhatsApp, and Skype). All interviews were audio-recorded with participant consent and transcribed verbatim an independent external agency.

### 2.4. Data Analyses

Content and thematic are argued to be legitimate data analysis techniques for qualitative descriptive analysis [62]. In this case, thematic analysis based on the six-phase framework put forward by Braun and Clarke [63, 64] was used, and the data analyzed concurrently with the data collection process by AM. To ensure that the results resonated with the target audience (i.e., medical practitioners, allied health professionals, specialist PD nurses, and healthcare administrators) and was in line with the study aims and methodology, a predominately “sematic or explicit” level approach to the analysis was taken [63, p. 84]. The transcribed data were first exported to NVivo (Version 14), de-identified using pseudonyms and checked against the audio-recoding for accuracy. The data were then reread to build an understanding of the content and identify any interesting and relevant aspects. This process also served to further refine the interview guide and identify areas of interest that may have needed deeper investigation in future interviews. Coding of the transcripts was undertaken using a predominately inductive, descriptive approach. Initial coding of each transcript began with annotation of the data during a process of close reading to highlight each concept and label as codes. During this process, codes were arranged into subcategories and categories (tentative themes) as they developed. A summary of each transcript was produced from the results of this process and sent to the corresponding participant to ensure that the contents of their interview were understood and interpreted correctly. Once the initial coding of all interviews was complete, the previously coded transcripts were revisited to ensure that their meaning had been fully explored, and the themes further refined to reflect the entire data set and address the study aims. The themes were then finalized and labeled according to their meaning. The results were reported descriptively with supportive data extracts. Debriefing with the research team was the method used to confirm interpretations and the development of themes.

### 2.5. Ethics

Ethical approval was obtained from the University of South Australia's Human Research Ethics Committee (ID: 205671). Participants received a Participant Information Sheet that outlined the purpose of the research, requirements for participation, data confidentiality, and contact information for various support organizations in case the interview caused any distress. It also stated that participation in the study was voluntary and that participants could withdraw at any time without consequence. Participants were also required to provide informed written consent prior to the interview.

## 3. Results

There were 18 participants (10 female) with a median age of 71 years (IQR 68-77). Geographically, most participants were from South Australia (six), followed by New South Wales (five), and Victoria (four). The remaining three participants were from Queensland, Western Australia, and the Australian Capital Territory. A majority (11) resided in metropolitan areas, and the remaining seven, regional Australia. Six participants identified symptoms consistent with stage 1 of the Hoehn and Yahr 5-point staging scale [65], six with stage 2, and six with stage 3. Almost all participants (14) indicted that for the treatment of PD a neurologist was their primary treating medical practitioner, three declared that they relied primarily on a neurologist/general practitioner combination, and only one, a general practitioner.

Thematic analysis of data resulted in four themes; (1) recommendations from trusted sources; (2) explorative experimentation and solution seeking; (3) intervention and service provider characteristics; (4) personal beliefs and abilities; and several subthemes (Table 1).

### 3.1. Theme 1: Recommendations From Trusted Sources

Participants in this study often reported that recommendations from external sources, especially those they viewed as “trusted”, had a substantial influence their pain management practices. They spoke of these sources primarily including clinician-led therapy and referrals, personal recommendations from support networks, and specialist health information.

#### 3.1.1. Subtheme: 1.1 Clinician-Led Therapy and Referrals

Recommendations from medical practitioners and allied health professionals were found to be the strongest influence on participants pain management practices. Participants described that this influence extended to the provision of interventions provided directly by the consulting clinician as well as informal and formal referrals. In total, 17 participants mentioned that clinician input had influenced at least one, and often many, therapeutic decisions. One participant, for example, described how their treatment journey for neuropathic pain had incorporated several clinical-led therapies and utilized the services of both medical practitioners and allied health professionals.

*“… I had several cortisone injections and then I went to a physiotherapist and after the cortisone injections I went to a pain management specialist who also gave me several injections in my spine, the nerve root, all the joints around the face of my spine. And then I saw another neurologist and he put me on gabapentin and pregabalin …”* (P5)

Furthermore, some participants reported that clinician recommendations were sufficient, on their own, to prompt them to initiate or change pain management practices.

*“… it didn't show anything, the CT scan, and he just prescribed amitriptyline 10 mg. Then it was still hurting, and I told the neurologist about it, and the neurologist said, “Oh, take 25 mg,” so I did.”* (P2)

#### 3.1.2. Subtheme: 1.2 Personal Recommendations From Support Networks

Seventeen participants mentioned the influence of family, friends, and other people with PD on their therapeutic decision-making. Participants mainly described how personal recommendations from support networks impacted their nonpharmacological treatment choices, including the type of intervention (such as osteopathy or physiotherapy) and the selection of providers.

*“I was sure that my neck would stiffen up and then I'd get a headache, and so I thought, “Well, I'll try a physiotherapist.” The first physiotherapist I went to didn't seem to help much. Friends swear by osteopaths, so I thought, “Well, I'll try an osteopath.””* (P2)

*“I went through three physiotherapists before I found him, and I found him because he was my daughter's friend, she knew him. She said, “Mum, you'll like the way he operates””* (P1)

However, in some instances, recommendations from support networks also influenced high-risk, life-changing therapies, especially when these recommendations came from other people with PD, whom participants often connected with through PD advocacy organizations. For instance, one participant discussed how the experiences of other people with PD influenced their decision to proceed with deep brain stimulation (DBS).

*“I joined a support group for Parkinson's, and two of the ladies in there had had DBS. And so I talked to them about it … a friend, put me in touch with a friend of hers who had DBS. I talked to him about it. I did quite a lot of research and decided it was well worth it.”* (P18)

#### 3.1.3. Subtheme: 1.3 Specialist Health Information

The use of specialist health information, primarily reliable health information websites, such as government sites, condition-specific sites, support organization sites, and medical journals, was also mentioned by some participants as influencing their pain management practices. Participants that accessed specialist health information described it as a supplementary influence rather than a primary one, using it to identify therapeutic options and providers. The main source of specialist health information discussed by participants was PD advocacy organizations. One participant, for example, discussed how they vetted therapeutic options identified through information from PD advocacy organizations with their general practitioner before using them.

*“… information that comes through from the different Parkinson's organizations … I usually run these things by my general practitioner and say, what do you think? And he'll either say, well, yes, we can try it …”* (P3)

Trust was vocalized by many participants as an important ingredient that affected the degree of influence that external sources had on their decision-making. The factors that promoted trust differed depending on the information source. For clinicians, participants indicted that they were more likely to trust a provider that takes time to explain the diagnosis and treatment options and is willing to answer questions.

*“It is a trusting relationship, built out of being treated like a human being, and sharing the information. Not saying, “I'm going to put you on this tablet, here's this prescription.” “I think we ought to use this medication because this is what it does. This is the symptoms that you're showing. I think this will work on this area. This is how it works, this is what it does in your brain,” etc. So the full open explanation and invitation for questions.”* (P8)

For family, friends, and other people with PD, trust was derived from the legitimacy participants attributed to these individuals because of a shared experience (i.e., PD and pain symptoms) and the perceived sincerity of their intentions. One participant, for example, when prompted why they seek therapeutic advice from other people with PD responded: *“Because I trust them, because we're all going through it together. They're not going to lie to me to make money. They're going to tell me their truth, and that's what I've really appreciated.”* (P7)

Finally, for specialist health information sources, such as PD advocacy organizations, participants described how trust was influenced by perceived source reliability and credibility.

*“When I was first diagnosed, he [neurologist] told me that reliable websites were by Parkinson's [organizations], which was Parkinson's Victoria and The Michael J Fox Foundation …”* (P18)

*“Other than the internet, where you've got to take a lot of that stuff with a grain of salt, I tend to stick to the bigger medical websites”* (P14)

In addition, therapeutic recommendations were spoken of by participants as having greater credibility when they were received from multiple sources. Exercise, for example, was often recommended to participants by several medical practitioners, allied health professionals, and other sources and was reported as a reason to initiate or continue to use that type of therapy. One participant, for example, emphasized how the consistent recommendations from multiple trusted sources reinforced their decision to pursue exercise and stretching as beneficial for managing their pain.

*“I suppose to some degree each of the pieces of advice have been reinforcing the other pieces. So, it seems to be certainly steering me down that exercise and stretching track … I've spoken to the neurologist, spoken to the GP, spoken to people that are also in the same sort of position. My wife and I, and two other couples run a Parkinson's peer support group … We've had guest speakers there, talking about pain and pain management and all sorts of aspects related to Parkinson's, and they seem to reinforce the views. I don't think there's any doubt that exercise is a good thing to do for people with Parkinson's.”* (P8)

### 3.2. Theme 2: Explorative Experimentation and Solution Seeking

Many participants reported that they had engaged in explorative experimentation and solution-seeking behavior at some point. These behaviors took different forms and included experimentation with nonprescription medications, modes of physical activity, nonpharmacological therapies, and novel treatment solutions.

Some participants described an active and conscious process of trial-and-error, moving from one failed therapy to another until they found a solution that provided a satisfactory level of pain relief.

*“… when I'm trying to first go to bed, my left foot starts to ache. And I've tried a cold-pack, cold-pack made it worse. I've tried a heat-pack, and it sort of made it a little bit better. But when I get in the shower and put the nozzle on as hot as I can possibly stand it, after a few minutes, it tends to settle it right down.”* (P14)

Other participants described a process of organic therapeutic development where coping strategies evolved into effective and permanent solutions.

*“I was in the normal laying in normal bed and barely could get up in the morning because the back was so sore. So about halfway through the night I'd go down and sit in the lounge chair and lay it back and found that I could get up and the back wasn't as sore, so we just went out and bought some bed that was up.”* (P12)

Importantly, in some cases, participants indicated that their explorative experimentation and solution-seeking behaviors were driven by a lack of clinician provided options, particularly from neurologists. Some participants, for example, reported that they had not been offered any pain management solutions, while others expressed frustration with the limited options that were suggested.

*“Well, because going to my neurologist didn't give me any other options really.”* (P7)

*“… Basically, he [neurologist] gave me nothing, really, to manage the pain, other than the anti-inflammatories.”* (P14)

### 3.3. Theme 3: Intervention and Service Provider Characteristics

There were several intervention and service provider characteristics that influenced participants' treatment practices. These included: therapeutic effectiveness; affective and ethical acceptability; and geographic accessibility.

#### 3.3.1. Subtheme: 3.1 Therapeutic Effectiveness

Therapeutic effectiveness—defined as the extent to which a therapy alleviated pain symptoms—was vocalized by 16 participants as a decisive factor in determining whether participants continued to use an intervention. Participants reported having very little tolerance for persisting with therapies that were not effective in reducing pain, and conversely, were eager to use therapies that provided relief. This influence extended across both pharmacological and nonpharmacological interventions, and even to medications prescribed by medical practitioners.

*“… my neurologist actually has given me another drug … which is supposed to be taken at night to possibly relax your legs. But I tried it, and it didn't do anything for me.”* (P13)

*“I usually have deep tissue [massage]. I find nothing else works, but it helps … It just goes to relieve the day-to-day stuff.”* (P17)

#### 3.3.2. Subtheme: 3.2 Affective and Ethical Acceptability

The affective and ethical acceptability of both the intervention and service provider was reported by 11 participants to influence their therapeutic choices, in some cases positively, and in others, negatively. When participants spoke positively about an intervention or provider, they typically focused on how it made them feel.

*“I've seen have been to a couple of exercise physiologists. The major one I've been going to … I think he's quite brilliant … he keeps the class lighthearted … He sets the tone for the class … And he does enough variety and exercises to make you feel like you've had a tiny bit of a workout and some days it's heavier than others. But I always walk out of there feeling more positive.”* (P11)

Conversely, when participants expressed negative views, they highlighted how the intervention or provider's characteristics conflicted with their values.

*“I know there's a lot of people who use marijuana for pain and that's something, growing up in the sixties, I don't think I'd want to know about it.”* (P3)

#### 3.3.3. Subtheme: 3.3 Geographic Accessibility

Geographic accessibility (how easily participants could reach the service provider) was both a barrier and enabler to pain therapy. For many participants, particularly those in regional areas, the distance and travel time made accessing specialist medical and allied health services challenging. Participants described having to forgo treatments altogether, select a provider that they viewed as inferior, or face delays in receiving treatment.

*“The PD warrior program is not available in our area. The closest is [redacted location], which is a three-and-a-half-hour drive for me …”* (P15)

*“Where I see my neurologist is two hours away in [redacted location]. There's a lot of physiotherapists up there and I could have gone to any of them, but I'm not going for a two-hour drive just to have a physiotherapy appointment. And it took a while to see the single physiotherapist that's in this town and he's just given me this range of exercises.”* (P6)

In contrast, participants reported a clear preference for health services that are in close geographic proximity to their residence. One participant, for example, noted the convenience of accessing health services through their retirement village provider and how it positively affected their pain management practices.

*“Since I've been here [retirement village], I've been going to their physiotherapists and having remedial massage at the wellness center which gives me some temporary relief”* (P4)

### 3.4. Theme 4: Personal Beliefs and Abilities

Personal beliefs and abilities encompassed several factors covering three key domains: participants beliefs about the positive and negative effects of the intervention; functional capacity and therapeutic tolerability; and experience, skills, and self-efficacy.

#### 3.4.1. Subtheme: 4.1 Beliefs About the Positive and Negative Effects of the Intervention

Beliefs about the effectiveness of therapies were mentioned by 11 participants as a reason for considering or dismissing certain therapies. Participants reported that evidence of therapeutic effectiveness was a key criterion on which pain management practice decisions were based. Participants noted that this evidence primarily came from past experiences and knowledge.

*“We had a little miniature sausage dog who had paralysis and had an operation. She had acupuncture every three months after that operation. And if you don't believe in acupuncture, if you just watch an animal's response … the result that it made for her …”* (P3)

*“I suppose there's never going to be a certainty with any therapy that is looking at pain issues, but I didn't have very much evidence that it was going to be a good idea, so didn't pursue it.”* (P8)

Concerns about the adverse effects of pharmacological interventions were voiced by 10 participants. Most participants voiced specific concerns, such as the risk of addiction (P6: *“I just didn't want to get addicted to them”*), while others shared a general negative sentiment towards pharmacological medications (P13: *“I try to avoid taking pills generally”*). Furthermore, the impact of participants concerns influenced treatment practices in varied ways. In most cases, they were only sufficient to modify or reduce usage, but in others, they resulted in participants refusing treatment.

*“The neurologist knows, he prescribed me levodopa and I had a struggle, I got the script filled, I looked at it and I just thought, “I cannot.” I understand how it works, that after so many years you either up it or you become dyskinetic, and I'm like, “I'm 60, I'm not doing this.””* (P1)

#### 3.4.2. Subtheme 4.2 Functional Capacity and Therapeutic Tolerability

Limitations in physical capacity, including fatigue, primarily because of the effects of PD but also from non-PD-related physical limitations, impacted some participants' ability to engage in certain modes of exercise therapy. One participant, for example, expressed a desire to exercise, motivated by the belief in its disease-modifying benefits, but faced challenges due to intermittent physical limitations and fatigue.

*“… I keep hearing that the best thing for Parkinson's is to exercise, but there's often things that stop me from exercising … I had a sore wrist, that was stopping me from gardening. When I had the knee problem, I had been walking once a week and that was up and down … The hardest thing I find is, I often won't feel good. This morning, I got up and I just felt lousy.”* (P2)

Similarly, therapeutic tolerability affected participants' ability or desire to adhere to the dose or intensity of therapy. One of the most prominent issues raised by participants was increased pain and discomfort from certain forms of exercise, and so too, albeit with less frequency, adverse reactions to pharmacological therapies.

*“I did go and try some [aqua] aerobics and I really didn't like that. I found that was too strong for me. Trying to use those weights in the water really stirred up a lot of pain. So I thought that's not a good thing to do for me.”* (P6)

*“… we tried the apomorphine pump and my body rejected that. It bruised terribly and I got really bad hives …”* (P15)

#### 3.4.3. Subtheme 4.3 Experience, Skills, and Self-Efficacy

Many participants developed skills and a sense of self-efficacy through previous experiences using therapies and interacting with healthcare providers that influenced their pain management practices. One participant, for example, discussed how undertaking exercise prior to their PD diagnosis facilitated ongoing engagement in similar forms of exercise for treatment of PD pain.

*“… it's been a part of our life and our makeup for a very long time. Exercise, nutrition, all of that. So yes, I believe that lifestyle probably has given me the mindset that I have at the moment.”* (P15)

Another participant noted that their previous work and personal experiences with the healthcare sector had given them the confidence to be proactive in their treatment practices.

*“I'm no doctor, I don't have that, but I've had a lot of experience with medication, with drugs, with doctors, with surgery … I don't mind advocating and being proactive.”* (P1)

## 4. Discussion

This study explored, from the patients' perspective, factors that influence pain management practices among people with PD living in Australia. A range of factors were reported by participants that were grouped across four themes: recommendations from trusted sources; explorative experimentation and solution seeking; intervention and service provider characteristics; and personal beliefs and abilities. These influencing factors were complex and interconnected, rarely operating independently. In addition, they often revealed gaps in pain care services and the challenges faced by people with PD in accessing appropriate care.

In this study, participants' pain management practices were largely shaped by recommendations from trusted sources, particularly medical practitioners and allied health professionals. Participants often relied on clinicians as a primary source for directly administering therapies as well as for recommendations and referrals to alternate treatment options. This finding is consistent with, and extends previous research, which have demonstrated the substantial role that clinicians play in shaping patients' treatment decisions across various other healthcare contexts [66–68]. Personal recommendations from support networks also influenced participants treatment practices, particularly with nonpharmacological therapies, and so too, albeit as a supplementary rather than primary source, specialist heath information. Importantly, participant access to these sources was often facilitated by PD advocacy organizations, through their support and educational services. Most previous research has focused on the influence of peer-support groups, online health resources, and other people's personal experiences on healthcare decision-making [69–71]. Our findings extend this literature by highlighting the crucial role that PD advocacy organizations play in bridging the gap between people with PD and reliable information sources. The importance of these organizations is underscored by the complex nature of PD pain and PD, making tailored information less accessible in the broader community. This emphasizes the need for continued support and development of these advocacy groups to enhance patient education and resources. Lastly, in agreement with previous research, trust was identified as an important determinant of the value that participants placed on recommendations from external sources [72–74]. The credibility of these recommendations was further enhanced when they originated from multiple sources.

Consistent with previous research [41], we found that while pain management practices were often clinician-led, participants also engaged in exploratory experimentation and solution-seeking behaviors characteristic of self-management, a widely recommended approach for treating chronic pain. In a review paper by Nicholas and Blyth [75], for example, investigating self-management strategies in chronic pain, the authors concluded there is sufficient evidence to show that individuals with chronic pain actively engaged in managing their condition experience less pain-related disability compared to those who rely on passive approaches, such as resting or relying on others. Additionally, guidelines published by Pain Australia and other peak bodies encourage patients to utilize nonpharmacological therapies that align with self-management practices [76, 77]. As such, research has largely focused on the factors influencing self-management. Findings indicate that clinician support—characterized by empathy and effective communication about pain management—is vital for achieving positive outcomes [78, 79]. However, we found that in some cases, participants' exploratory behaviors were motivated more by a lack of clinician provided therapeutic options, particularly from neurologists, rather than by clinician self-management support. This highlights a substantial evidence practice gap in the provision of pain care services for people with PD. Clinician barriers to providing self-management support to patients may include: time limitations of the clinical visit [80, 81]; lack of appropriate clinical knowledge and referral networks [80–82]; difficulties discussing the biopsychosocial approach to pain management [81]; and difficulties diagnosing and treating the variety of PD pain syndromes [83, 84]. These findings suggest that enhancing clinician support and addressing barriers could significantly improve pain management outcomes for people with PD.

The geographic accessibility of interventions and service providers was an influencing factor identified in this study that is worthy of note. We found that participants residing in regional Australia faced geographic challenges accessing timely and appropriate specialist medical and allied health services for the management of pain, which adversely affected their level of care. These findings are supported by previous research in the broader PD population that has found accessibility of a range of healthcare services in several jurisdictions, including Australia, poses a barrier for people with PD [85]. In Australia, regional populations are often dispersed across large distances and located far from urban centers with concentrated medical services making geographic accessibility a prevalent issue in the Australian healthcare system [86]. In a cross-sectional study investigating the provision of health services by patients with PD in Victoria by Lubomski et al. [87], the authors found that almost 20% of regional clinic participants had to travel more than 100 km one-way to access allied healthcare services. The challenges faced by people with PD who experience pain in accessing suitable healthcare services are especially severe, as many experts believe that a multimodal approach—combining pharmacological and nonpharmacological therapies—and a multidisciplinary approach involving various specialists are necessary to tackle the issue [88, 89]. Despite efforts by the Australian government, such as the Stronger Rural Health Strategy that aims to build a sustainable, high-quality health workforce in regional Australia [90], our results suggest that for people with PD living in regional Australia, appropriate pain management services are still lacking. One potential solution to these access challenges is highlighted in a recent study by Sharrad and Harmon [91], which evaluated the implementation of a regional interdisciplinary care model. In this model, four clinical academics, representing nursing, physiotherapy, occupational therapy, and pharmacy, conducted a joint, three-hour consultation with a person with PD (and their carer, where applicable). The results were positive, indicating that this approach may offer a feasible and effective model for delivering comprehensive care in regional settings.

This study also identified several factors related to personal beliefs and abilities that modulated participants' pain management practices. Among these factors, participants' capacity to engage in, and ability to tolerate certain forms of exercise therapy are of particular interest and relevance. Exercise is often recommended for people with PD due to its overall health benefits, potential to modify the disease, and its effectiveness in managing disease-specific symptoms, including pain [92–95]. Indeed, in this study, we also found that exercise was frequently recommended for the management of pain. However, participants often reported that they were unable to engage in their preferred mode or dose of exercise due to functional limitations, other health issues, or exercise-induced pain. These findings are supported by, and extend, previous research that has found similar barriers to exercise within the broader PD population [96, 97]. Factors contributing to these challenges may include the multifactorial nature of PD pain pathophysiology and presentation [28–31], the disease's prevalence among older adults—over 60% of PD patients in Australia are aged 65–84 years [2]—and the absence of specific exercise guidelines for managing pain in PD [98]. This research, therefore, underscores the urgent need for the development of exercise guidelines specifically tailored to pain management in PD to encourage ongoing participation.

A strength of this study was the diversity of recruitment of participants from across Australian states and different localities (such as metropolitan and regional) which contributed to the richness of the data collected. Having an interview guide that was developed through consultation with the research team with diverse skills and expertise contributed to the depth of data collected. This research was also informed by and reported based on best practice standards in the conduct and reporting of qualitative research.

However, this research does have limitations. Although efforts were made to recruit a diverse sample, our participant cohort did not include, according to the Hoehn and Yahr five-point staging scale [65], those with advanced-stage PD, which may limit the applicability of the findings to those in early- and mid-stage PD. Additionally, while this research provides rich information about the factors influencing pain management from the perspective of people with PD, it does not include the perspectives of clinicians. Further research with this stakeholder group is warranted. In addition, although participants were encouraged to reflect broadly on the factors influencing their pain management practices, including both pharmacological and nonpharmacological approaches, the interview guide did not include questions about specific therapies, such as dopaminergic medication. Given that dopaminergic therapy can alleviate certain PD-related pain, especially pain associated with motor fluctuations or dystonia, this may have influenced participants' experiences and responses [99]. As a result, insights into the factors influencing specific therapies may be limited and warrant further investigation. Future research could also explore cross-cultural and international comparisons to better understand how healthcare systems, professional roles, and cultural expectations influence pain management in PD.

It is important to acknowledge that during the analysis process, some overlap between themes emerged. This crossover reflects the complex and interconnected nature of the factors influencing pain management practices in people with PD. For instance, exercise was frequently recommended to participants by a variety of trusted sources (Theme 1) and aligns with explorative and solution-seeking behaviors (Theme 2). However, some participants also reported difficulties engaging in exercise due physical limitations (Sub-theme 4.2). While every effort was made to maintain thematic clarity, these overlaps were intentionally preserved to reflect the real-world complexity of pain management. Rather than a limitation, this interconnectedness adds richness to the data and should be considered when interpreting the findings.

In conclusion, this study extends the existing literature by providing insight and understanding into the factors that influence pain management practices from the perspectives of people with PD set within the context of the Australian healthcare system. The findings shed light on the complex nature of therapeutic decision-making, underscoring the interaction between external and internal influences while revealing notable gaps in the provision of PD pain care services. While clinicians play a critical role in the management of pain in people of PD, this study highlights the importance of recognizing and supporting the contributions of patients, carers, and the wider community networks in managing pain. Effective strategies require collaborative engagement across all stakeholders, patients actively participating in care, families, and communities providing support, and multidisciplinary clinics fostering integrated and person-centered approaches. Strengthening these connections may enhance the pain management practices for people with PD.

## Figures and Tables

**Table 1:** Factors influencing pain management practices.

Themes	Subthemes
Recommendations from trusted sources	Clinician-led therapy and referrals
	Personal recommendations from support networks
	Specialist health information
Explorative experimentation and solution seeking	n/a
Intervention and service provider characteristics	Therapeutic effectiveness
	Affective and ethical acceptability
	Geographic accessibility
Personal beliefs and abilities	Beliefs about the positive and negative effects of the intervention
	Functional capacity and therapeutic tolerability
	Experience, skills, and self-efficacy

## Data Availability

The data that support the findings of this study are available from the corresponding author upon reasonable request.
